# Fetal Down syndrome screening models for developing countries; *Part I: Performance of Maternal Serum Screening*

**DOI:** 10.1186/s12913-019-4446-x

**Published:** 2019-11-27

**Authors:** Chanane Wanapirak, Wirawit Piyamongkol, Supatra Sirichotiyakul, Fuanglada Tongprasert, Kasemsri Srisupundit, Suchaya Luewan, Kuntharee Traisrisilp, Phudit Jatavan, Theera Tongsong

**Affiliations:** Department of Obstetrics and Gynecology, Faculty of Medicine, Chiang Mai University, Chiang Mai, 50200 Thailand

**Keywords:** Down syndrome, Prenatal screening, Prenatal diagnosis, Cost-benefit, Developing country

## Abstract

**Background:**

To identify the performance of fetal Down syndrome (DS) screening for developing countries.

**Methods:**

A prospective study on MSS (maternal serum screening) with complete follow-ups (*n* = 41,924) was conducted in 32 network hospitals in the northern part of Thailand. Various models of MSS were tested for performance.

**Results:**

MSS based on Caucasian reference range resulted in very high false positive rate (FPR; 13%) in our country, compared to the rate of 7.8% with our own (Thai) reference range, whereas the detection rate was comparable. As individual screening, C-S (contingent first trimester screening including PAPP-A, and free beta-hCG, classified as a) high risk [> 1:30], indicated for invasive diagnosis; b) intermediate risk [1:30–1500], indicated for STS; and c) low risk [< 1:1500], need no further tests.) was the most effective model (sensitivity 84.9%, FPR 7.7%) but nearly one-third needed the second trimester test (STS) because of intermediate results. Additionally, about one-third had their first visits in the second trimester and had no chance of FTS (first trimester screening). C-S plus STS had a sensitivity of 82.4% and FPR 8.1% whereas independent first and second trimester screening model (I-S) gave the sensitivity of 78.4% and FPR of 7.5% but was much more convenient and practical.

**Conclusion:**

C-S plus STS was the most effective models while I-S model was also effective and may be better for developing countries because of its simplicity and feasibility.

## Background

Prenatal screen of Down syndrome (DS) with maternal serum screening (MSS) has been established for two decades [1], leading to a decrease in the prevalence of DS in many developed countries. However, the prevalence in countries with low-resource settings has not changed much in recent years. Soon, we will be launching the implementation of DS screening for all women as a national policy, free of charge. It is important that before such implementation, the effectiveness of the screening methods must be thoroughly evaluated. It may be different from that used in other parts of the world, as indicated by studies in developing countries. In our experiences, such published effectiveness is not reproducible in developing countries. For example, the average body size of Thai women is much smaller than that of women in the western countries, whereas the fetal size is similar. The serum biomarkers derived from the fetus are diluted in maternal blood volume of much different size, causing great difference of concentrations in maternal blood. Many studies have shown that a racial factor has an impact on serum biomarker levels, resulting in less reliable screening accuracy [2–6]. The MSS concentrations are significantly higher among Asian pregnant women [7–9], even after correction with maternal size [7, 10, 11]. Correction for ethnicity can be achieved by either using medians of serum biomarkers for specific ethnicity [7] or using ethnic factor to correct the truly measured levels in any ethnic population to equivalent levels for a Western woman [12]. For example, ethnic factor of b-hCG level for Asian women is 0.84, used to multiply by the true measured levels before calculation for MoMs and interpretation based on the western reference ranges. Certainly, the former method is preferred. Ethnic factors for correction used in most machines are not accurate when used in other geographical areas. For instance, among Thai women, false positive rate of serum screen corrected with ethnic factor is as high as approximately 12% [13], instead of 5% as most commonly recommended. Such a high false positive rate leads to a great number of invasive diagnoses, exceeding our capability of chromosome investigations, thus precluding universal screening as a national policy. Therefore, the more proper strategy needs to be developed. This study did not include the techniques that are not practicable in low resource settings, such as NT (nuchal translucency), which needs expertise and is not widely available; integrated MSS test, which needs two screenings and the costs are double. The purpose of this study is to determine the performance of various models of MSS among Thai pregnant women representing those in developing countries.

## Methods

A study of prospective screening / diagnostic test was conducted with ethical approval by the institutional review board and the women were recruited with written informed consent. The hospital network was developed in the northern part of Thailand, including 33 community hospitals, together with development of the counseling teams, ultrasound standardization for gestational age, maternal blood sampling/collection and logistic system. The study population comprised women attending antenatal care at the network hospitals between September, 2011 and May, 2016. The inclusion criteria were 1) Thai ethnicity, 2) singleton pregnancy, and 3) attending antenatal care clinic before 20 weeks of gestation. The exclusion criteria were 1) fetal anomaly, 2) incomplete data, and 3) serum samples that were collected and transported to the laboratory for more than 24 h.

### Steps in the research process

The baseline demographic data of the women were assessed and immediately recorded in the protocol form at the time of blood sample collection. The data included maternal age, parity, body weight, ethnic origin and smoking habits or medical complications. All women received pretest counseling by the counselor teams specifically trained and standardized for prenatal counseling in this project. The collected blood samples were immediately transferred to the laboratory and were centrifuged for serum separation. All serum samples were measured for biomarkers at the study center using the same laboratory (fully-automated immunoassay, using DELFIA® Xpress system; Perkin Elmer, Waltham, MA, USA) and standard assay screening kits of serum biomarkers (PAPP-A, AFP, beta-hCG, and uE3). All assays were performed in batches to eliminate inter-assay variations.

### Risk determination

The risk category was based on the screening in real practice, 1) STS (second trimester screening) at 15–20 weeks for women going for their first visit in the second trimester, including AFP, free beta-hCG, and unconjugated estriol, using cut-off of 1:250 for high risk; and 2) Contingent first trimester screening (C-S) for women going for their first visit at 10–14 weeks, including PAPP-A, and free beta-hCG, classified as a) high risk (> 1:30), indicated for invasive diagnosis; b) intermediate risk (1:30–1500), indicated for STS; and c) low risk (< 1:1500), need no further tests. The high risk women were referred for invasive diagnoses (amniocentesis) at the study center. Risk determination of the screening was based on the Caucasian reference ranges (built-in). The gestational age was based on ultrasound biometry of the crown-rump length in the first trimester or biparietal diameter/head circumference in the first half of pregnancy.

### Follow-up of pregnancies

All recruited women were followed-up for pregnancy complications such as abortion, preterm labor, intrauterine growth restriction, pregnancy-induced hypertension, antepartum hemorrhage, intrapartum and postpartum complications. Fetal loss related to diagnostic procedures was also evaluated for later use in cost-benefit analysis. All newborns were prospectively assessed by the neonatologists / pediatricians in the team of researchers. Neonatal chromosome study was performed only for the fetuses clinically suspected of chromosomal abnormalities after evaluation by the neonatologists. Diagnosis of fetal DS was based on chromosome studies by chorionic villous sampling, amniocentesis, or postnatal studies, while non-DS was based on chromosome studies or the conclusion by the neonatologists in cases of absence of chromosome study results.

### Definition of primary screening models

In real practice, the patients were managed using the screening based on Caucasian reference ranges (CRR) as mentioned above. However, the data permitted us to re-categorize the primary screening into several models, *using Thai reference ranges (TRR)* [14, 15], as follows: 1) Maternal age alone: High risk if maternal age is up to 35 years or more; 2) STS (second trimester screening) alone: The risk derived from serum levels of AFP, free b-hCG and uE3; 3) FTS (first trimester screening): The risk derived from serum levels of PAPP-A and free b-hCG; 4) Contingent FTS screening (C-S): The risk derived from serum levels of PAPP-A and hCG) and categorized into three groups according to the risk as mentioned above; 5) C-S plus STS: Contingent FTS for women with first visit in the first trimester and STS alone for women with first visit in the second trimester; 6) Independent FTS and STS (I-S): FTS alone for women with first visit in the first trimester and STS alone for women with first visit in the second trimester.

### Statistical analysis

The diagnostic performance (detection rate and false positive rate) was assessed for the various models mentioned above. Sample size estimation was based on previous studies [16–19], which reported that MSS had a sensitivity of ≥70% at a false positive rate of 5% for fetal DS screening among unselected pregnant women. Given a confidence level of 95% and acceptable error in diagnosis of 0.1, the project needed at least 72 fetuses with DS. The prevalence of fetal DS is about 1:600 at gestational age of 16 weeks. Therefore, the study needed a sample size of at least 43,200 tests. Statistical analysis was performed using IBM SPSS version 21.0(IBM SPSS Statistics for Windows, Released 2012. Armonk, NY: IBM Corp).

## Results

**Fig. 1:**
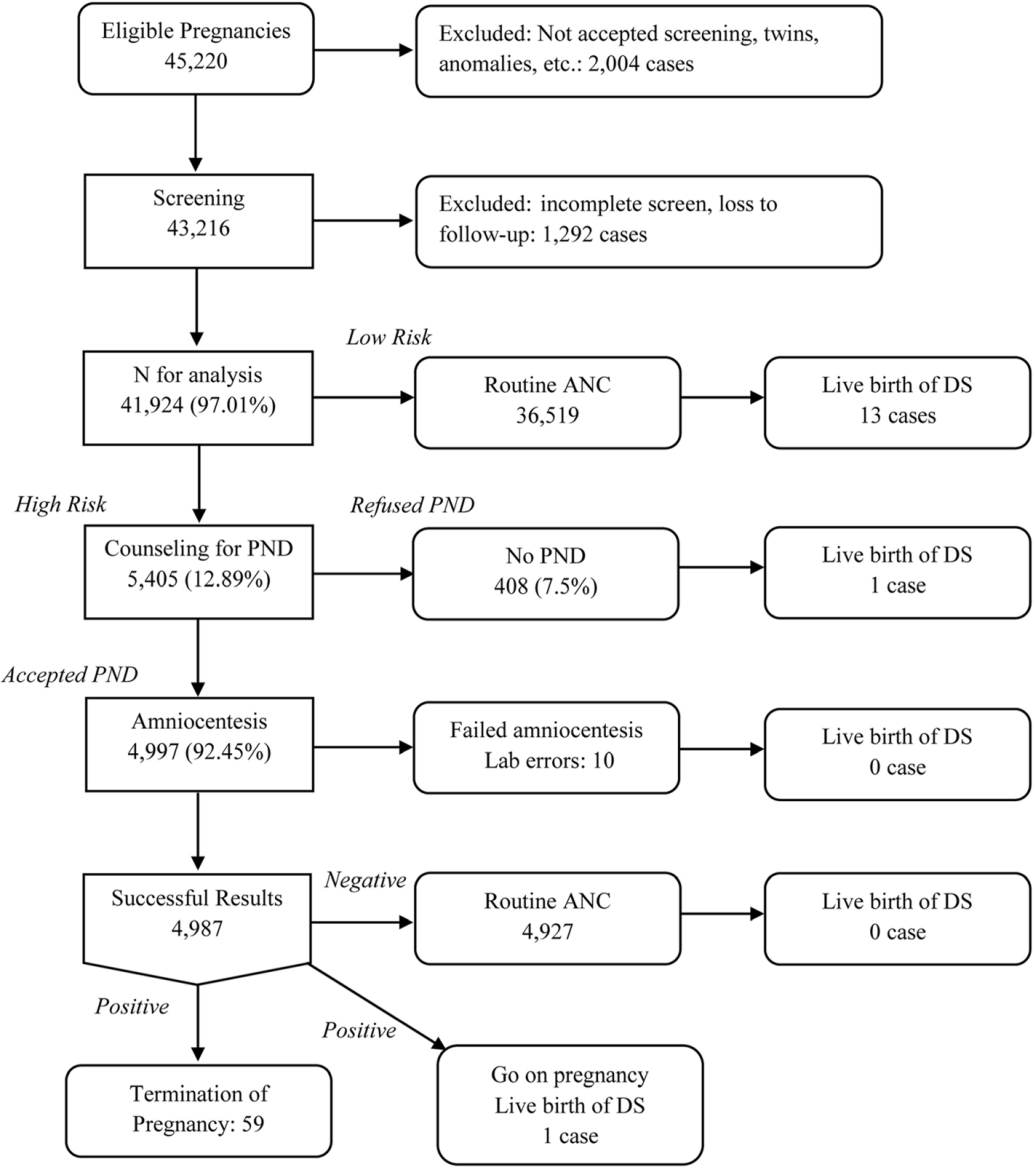
Flow chart of screening cascades: contingent FTS plus STS based on Caucasian reference ranges with ethnic (Asian) factor correction

Of 45,220 eligible pregnancies, 43,216 attended antenatal care clinics and met the inclusion criteria. Of them, 41,924 women accepted MSS (C-S plus STS based on CRR) either in the first trimester contingent screening or second trimester and complete data of final outcomes as shown in Fig. 1. Of all, 5405 (12.9%) were categorized as high risk (HR), including 4997 (92.45%) undergoing amniocentesis and 408 (7.55%) doing nothing. Of all, 74 pregnancies had fetal DS, including 61 and 13 with and without prenatal detection, respectively. The prevalence of fetal DS was 0.18% or 1:567. Of non-affected cases, spontaneous fetal loss after 16 weeks was 59/36,927 (0.16%) and fetal loss rate among women undergoing amniocentesis was 33/4997 (0.66%). *(Very high acceptability was observed in all steps, due to free of charge in this project).*

About two-thirds of women (29,692; 70.8%) first attended antenatal care in the first trimester and had a chance of FTS and C-S whereas the remaining nearly one-third (12,232; 29.2%) had first visits in the second trimester and could undergo only STS.

Screening performance of the various models is shown in Table 1. Of note, screening (C-S plus STS) based on the CRR has the comparable detection rate when compared to that based on TRR (86.8% vs 84.9%; 76.2% vs 76.2 and 83.8% vs 82.4% for C-S, STS and C-S plus STS, respectively). However, the false positive rate was much higher with the CRR when compared to that based on TRR (13.7% vs 7.7%; 11.0% vs 9.2 and 12.9% vs 8.1% for C-S, STS and C-S plus STS, respectively).

**Table 1:** Summary of the primary screening performance for fetal Down syndrome

	Final Risk	*N* (%)	Down Syndrome	Sensitivity	False Positive
Maternal Serum Screening Based on Caucasian Reference Ranges					
Contingent screen (C-S)	LR	25,580 (86.2%)	7	86.8%	13.7%
	HR	4112 (13.8%)	46		
	Total	29,692 (100%)	53		
Second trimester serum screen (STS) alone	LR	10,877 (88.9%)	5	76.2%	11.0%
	HR	1355 (11.1%)	16		
	Total	12,232 (100.0%)	21		
C-S plus STS	LR	36,457 (87.0%)	12	83.8%	12.9%
	HR	5467 (13.0%)	62		
	Total	41,924 (100%)	74		
Maternal Serum Screening Based on Thai Reference Ranges					
Contingent screen (C-S)	LR	27,361 (92.1%)	8	84.9%	7.7%
	HR	2331 (7.9%)	45		
	Total	29,692 (100%)	53		
Second trimester serum screen (STS) alone	LR	11,093 (90.7%)	5	76.2%	9.2%
	HR	1139 (9.3%)	16		
	Total	12,232 (100%)	21		
C-S plus STS	LR	38,454 (91.7%)	13	82.4%	8.1%
	HR	3470 (8.3%)	61		
	Total	41,924 (100%)	74		
First trimester serum screen (FTS) alone	LR	27,627 (93.0%)	11	79.2%	6.8%
	HR	2065 (7.0%)	42		
	Total	29,692 (100%)	53		
I-S (Independently combined first and second trimester)	LR	38,720 (92.4%)	16	78.4%	7.5%
	HR	3204 (7.6%)	58		
	Total	41,924 (100%)	74		
Age-based Screening					
Combined First and Second Trimester	LR	36,967 (88.2%)	52	29.7%	11.8%
	HR	4957 (11.8%)	22		
	Total	41,924 (100%)	74		

Of all screening tests using TRR, FTS had better screening performance than STS (detection rate of 79.2% vs 76.2%, and false positive rate of 6.8% vs 9.2%, respectively). Nevertheless, C-S had a significantly higher detection rate when compared with simple FTS (84.9% vs 79.2%; Chi-square test; *p*-value < 0.001); with slightly higher false positive rate (7.7% vs 6.8%; Chi-square test; *p*-value < 0.001).

Among C-S, the actual risk based on CRR was classified as low risk (LR), intermediate (IR) and high risk (HR); 18,336 (61.8%), 10,962 (36.9%) and 394 (1.3%), respectively. However, when reclassified using TRR, LR, IR and HR were: 23,314 (78.5%), 5984 (20.2%), and 394 (1.3%), respectively. Note that all cases classified as LR and HR by CRR were still classified as LR and HR by TRR but, importantly, more than one-third of IR by CRR were reclassified as LR when using TRR. Interestingly, with TRR the screening performance was much better as indicated by a marked decrease in false positive rate (13.7% vs 7.7%) while the detection rate was nearly the same. Likewise, STS based on CRR had also a significantly higher false positive rate than STS based on TRR with the same detection rate (McNemar Chi-square test; *p*-value < 0.001) as shown in Table 1. Age-based screening gave the lowest detection rate (29.7%) with a relatively high false positive rate (11.8%).

In summary, a) Maternal serum screening (without NT) based on CRR had a very high false positive rate when compared to that based on TRR, b) As an individual test, C-S was the most effective serum screening model (detection rate of 84.9% with false positive rate of 7.7%). c) In real practice, C-S plus STS gave the best screening performance (detection rate of 82.4% with false positive rate of 8.1%), d) Age-based screening had the lowest detection rate (29.7%) and relatively high false positive rate (11.8%).

## Discussion

The important insights gained from this study are: 1) CRR for maternal serum screening used in other parts of the world may probably lead to erroneously high false positive rate, resulting in excessive burden of amniocentesis and unnecessary fetal losses. 2) Theoretically, C-S was the most effective serum screening test. 3) In real practice, C-S plus STS gave the best screening performance since not all women were able to undergo FTS, nearly one-third having first visits after first trimester. Nevertheless, I-S is a more practical model in terms of patient’s convenience of first visit timing and only once screening.

Interestingly, two very unusual findings were observed in MSS using CRR: 1) very high rate of intermediate risk of FTS, more than 30% (but 20% when using Thai reference range), and 2) high false positive rate in both FTS and STS. Because of the unacceptably high false positive rate and intermediate risk rate of MSS using CRR, we strongly recommended reference ranges of its population instead of ethnic correction factor unless it has been proven to be accurate in large sample size in its own population. Importantly, the very high rate of amniocentesis secondary to false positive rate is not only associated with the great number of fetal losses but the burden of chromosome laboratories is also too problematic for the government to include DS screening in health coverage as a national policy. Finally, the performance derived from our own reference range would be better used to base cost-benefit analysis (CBA) on subsequently. Cost-benefit directly depends on the performance of the screening test, both sensitivity and specificity. The accuracy of diagnostic performance is very important for further evaluation of CBA. The sensitivity and specificity of the screening test must be based on the real practice. It directly determines the number of amniocenteses and non-invasive prenatal tests (NIPT) or cell-free fetal DNA technique.

Considering the best model for developing countries, several aspects must be taken into account: feasibility, expertise requirement, simplicity, costs of screening tests and invasive diagnosis, capacity in chromosome lab development etc. Note that this study did not include integrated tests, because of the high costs of double screenings with small additional detection rate. It also excluded NT and genetic sonogram, because of the need for high expertise, not practical for extensive use in low resource settings. FTS alone was not suitable since many women had their first visit in late gestation. C-S plus STS was most effective but had higher costs due to the high rate of intermediate risk requiring STS and was complicated by counseling as well as anxiety during waiting for the final risk. Therefore I-S seems to be more attractive, though with slightly lower detection rate.

### Strength

The strengths of this study are as follows: 1) It is a prospective large-scale population-based study. 2) All models were based on feasibility and simplicity. 3) All newborns, either high risk or low risk determined by MSS, were evaluated for DS by pediatricians in the project. 4) All samples were properly collected and transported and run at the same laboratories. We were conscious of the logistics and temperature, which have been proven to have an obvious influence on the serum marker levels, as suggested by our preliminary study [20]. 5) The high homogeneity of the participants (Thai ethnicity). 6) This project was undertaken under the support of a non-profit organization without conflict of interest.

### Weakness

The weaknesses of this study are as follows: 1) Some other well-known strategies like integrated screening or fully combined first trimester screening were not included; however, such strategies are not suitable for low resource settings. 2) Trisomy13 and 18 were not taken into account since they were not a major problem in developing countries and were considered incompatible with life. 3) The uptake rate of MSS in this study could not represent the real practice since all women in this project were offered the MSS free of charge. 4) The dataset originally used for categorization based on CRR could not be exactly the same as that based on TRR since the cases categorized as low risk and high risk by CRR in the first trimester did not contribute data for the second trimester screening, whereas the intermediate risk women did. In principle, if they were firstly categorized using TRR, those women might have become intermediate risk with contributing data for second trimester screening. 5) The models in this study were primarily focused on our national health care. Thus, the results might not be perfectly accurate for other countries’ strategies. However, we believe that this could probably be a model for several developing countries especially many parts of Asia.

## Conclusion

MSS reference ranges derived from Caucasian pregnant women could not be effectively used with Southeast Asian women even with the use of racial factor correction. This is because the false positive rate is far too high. While the detection rate is comparable, the rate of amniocentesis (false positive) is high, leading to an increased burden of amniocentesis and chromosome laboratories as well as high fetal loss rate secondary to the procedure. Each geographical area should have its own reference ranges for its own population.

## Data Availability

The datasets analyzed during the current study are available from the corresponding author upon reasonable request.

## Abbreviations

AFP: Alpha-fetoprotein
CBA: cost-benefit analysis
CRR: Caucasian reference ranges
C-S: contingent first trimester screening
DS: Down syndrome screening
FPR: false positive rate
FTS: First trimester screening
hCG: human chorionic gonadotropin
HR: High risk
I-S: independent first and second trimester screening
LR: Low risk
MSS: Maternal serum screening
NIPT: non-invasive prenatal test
NT: Nuchal translucency
PAPP-A: Pregnancy-associated plasma protein A
STS: Second trimester screening
TRR: Thai reference range
uE3: unconjugated estriol
